# Multimorbidity, health Literacy, and quality of life among older adults in an urban slum in India: a community-based cross-sectional study

**DOI:** 10.1186/s12889-024-19343-7

**Published:** 2024-07-09

**Authors:** M. Yogesh, Naresh Makwana, Nidhi Trivedi, Naresh Damor

**Affiliations:** Department of Community Medicine, Shri M P Shah Government Medical College, Jamnagar, Gujarat India

**Keywords:** Multimorbidity, Older adults, Urban slums, Quality of life, India

## Abstract

**Background:**

India is experiencing a rising burden of chronic disease multimorbidity due to an aging population and epidemiological transition. Older adults residing in urban slums are especially vulnerable due to challenges in managing multimorbidity amid deprived living conditions. This study aimed to assess the prevalence of multimorbidity, associated health literacy, and quality of life impact in this population.

**Methods:**

A community-based cross-sectional study was conducted among 800 adults aged ≥ 65 years in an urban slum in Gujarat, India. Data on sociodemographics, physical and mental health conditions, health literacy (HLS-SF-47), quality of life (Short Form-12 scale), and social determinants of health were collected. Multimorbidity is ≥ 2 physical or mental health conditions in one person.

**Results:**

The prevalence of multimorbidity was 62.5% (500/800). Multimorbidity was significantly associated with lower physical component summary (PCS) and mental component summary (MCS) scores on the SF-12 (*p* < 0.001). After adjusting for sociodemographic variables, the odds ratio of 0.81 indicates that for every 1 unit increase in the health literacy score, the odds of having multimorbidity decrease by 19%. Older age within the older adult cohort (per year increase) was associated with greater odds of multimorbidity (AOR 1.05, 95% CI 1.02–1.09). Physical inactivity (AOR 1.68, 95% CI 1.027–2.77) and lack of social support (AOR 1.57, 95% CI 1.01–2.45) also increased the likelihood of multimorbidity.

**Conclusion:**

There is a substantial burden of multimorbidity among urban slum dwellers aged ≥ 65 years in India, strongly linked to modifiable risk factors like poor health literacy and social determinants of health. Targeted interventions are essential to alleviate this disproportionate burden among urban slum older adults.

**Supplementary Information:**

The online version contains supplementary material available at 10.1186/s12889-024-19343-7.

## Introduction

Multimorbidity, defined as the coexistence of two or more chronic conditions in one person, is increasingly common among older adults globally [1]. The prevalence of multimorbidity increases with age and is greater in low- and middle-income countries than in high-income countries [2]. A multi-country population-based study stretching across low-, middle-, and high-income countries found that the prevalence of multimorbidity increases with age [3]. However, there was heterogeneity in the estimates based on setting, participant age group, and the number and type of chronic conditions included.

In India, the burden of multimorbidity is expected to rise dramatically due to the rapidly aging population coupled with the epidemiological transition from communicable to noncommunicable diseases [4]. Studies from both urban and rural parts of India have shown a high prevalence of multimorbidity among older adults, ranging from 55 to 83% [5, 6]. The most common chronic disase included hypertension, diabetes, heart disease, chronic respiratory conditions, musculoskeletal disorders, and mental health conditions.

With the accumulation of multiple chronic conditions, older adults are at increased risk of adverse health outcomes, including declines in physical and cognitive functioning, poor quality of life, and increased healthcare utilization [7–9]. Multimorbidity has been associated with lower health-related quality of life across different populations [10, 11]. An impaired quality of life leads to a loss of independence, social isolation, and greater demands on family members as caregivers [12].

In India, there are wide urban-rural and socioeconomic disparities in access to healthcare and social support systems for older adults with multimorbidity. Those living in urban slums are especially vulnerable due to poverty, substandard housing, lack of infrastructure, and barriers to healthcare access in these informal settlements [13]. The challenges of managing multimorbidity are greater for slum dwellers because of high out-of-pocket expenditures for health services and medications [14].

Despite the growing size of this vulnerable population, there is limited community-based data on the burden and impact of multimorbidity among older adults in urban slums in India. Most related studies have been conducted in community or hospital settings, with an underrepresentation of urban slum populations. Given their deprived living conditions and lack of social protection for healthcare, older slum dwellers likely experience a disproportionately greater burden of multimorbidity and related adverse consequences.

There is a need for representative data on the prevalence of multimorbidity and its relationship with health-related quality of life in this urban slum population. This approach can help identify high-risk groups and modifiable factors to inform targeted interventions and appropriate health services for multimorbidity management.

Health literacy, defined as the degree to which individuals can obtain, process, and understand basic health information needed to make appropriate decisions, is an important factor in the prevention and management of chronic diseases. However, there is limited data on the association between health literacy and multimorbidity burden, especially among vulnerable populations like urban slum dwellers in India.

Therefore, the present study aimed to (i) assess the prevalence of multimorbidity among older adults living in an urban slum, (ii) examine the association of multimorbidity with health literacy and quality of life in this population, (iii) identify high-risk groups based on sociodemographic factors. We hypothesized that multimorbidity would be highly prevalent in this population and associated with poor health literacy and quality of life. This is the first study, to our knowledge, that aims to assess health literacy related to multimorbidity among urban slum dwellers aged ≥ 65 years.

## Methodology

### Study design and setting

This was a community-based cross-sectional study conducted in an urban slum located in Gujarat between April 2023- Dec2023. This slum has a population of approximately 50,000 residing across 20 municipal wards. The residents belong predominantly to lower socioeconomic status groups and face challenges such as poverty, inadequate housing, poor sanitation, and limited access to healthcare service.

### Sample size calculation

Considering a prevalence of 45% based on previous Indian studies [14], with 5% absolute precision and a design effect of 2 for the multistage sampling, the sample size was calculated using the formula:

n = Z2*P(1-P)/d2 *design effect

Where, Z = 1.96 at 95% confidence interval *P* = 45% = 0.45, d = 5% = 0.05 Design effect = 2

Plugging in the values: n = (1.96)2 × 0.45(1-0.45) / (0.05)2 × 2 *n* = 768, the final minimum sample size was estimated rounded up to be 800.

The sampling technique employed was a multistage random sampling approach. In the first stage, four out of the approximately 20 municipal wards in the urban slum area were randomly selected using the lottery method. Subsequently, systematic random sampling was applied in each of the four chosen wards to select households. A rough sketch map was used to guide the process, with every 5th household included in the sample. In the third stage, within each selected household, eligible individuals (aged ≥ 65 years) were listed, and one older adult was randomly chosen using the lottery method.

If the initially selected participant was not available at home after two visits, the next older adult from that household was approached. If no eligible individual was available in the selected household, the adjacent household was approached using the lottery method again.

The eligibility criteria included age ≥ 65 years, residence in the selected households, and providing informed consent, while the exclusion criteria included inability to communicate, bedridden, or unwilling to participate.

### Data collection

In this research endeavor, a meticulously designed pretested interviewer-administered questionnaire served as the primary tool for data collection. The questionnaire elicited sociodemographic information from participants, providing insights into the studied population. Additionally, participants self-reported chronic conditions, providing information on health issue prevalence. The chronic conditions elicited included hypertension, diabetes, heart disease, stroke, chronic respiratory diseases, musculoskeletal disorders, neurological disorders, mental health conditions, cancer, and others. The questionnaire tool used to collect this data was adapted from validated instruments used in previous Indian and global studies [15] on multimorbidity. It was pretested in a pilot study for comprehensibility, validity, and reliability before use in this study. We have provided the final questionnaire as a supplementary file 1.

The Short Form-12 (SF-12) assessed health-related quality of life [16]. This widely recognized instrument evaluates physical and mental health. Furthermore, the 47-item Short Form Health Literacy Scale (HLS-SF-47) was administered to measure participants’ health literacy across different domains [17, 18]. Anthropometric data such as height, weight, and blood pressure were also recorded. Physical activity level: Assessed using the Global Physical Activity Questionnaire (GPAQ) [19]. Smoking status: Categorized as a non-smoker, current smoker, or former smoker. Alcohol use: Drinking frequency and number of drinks per occasion. Dietary patterns: Assessed using a food frequency questionnaire [20]. Social support/living arrangements: Measured using the Multidimensional Scale of Perceived Social Support (MSPSS) [21]. Healthcare access: Based on the distance to the nearest health facility and reported barriers to healthcare. (Limited healthcare access: Defined based on the distance to the nearest health facility (> 5 km) and self-reported barriers to healthcare access including lack of transportation, inability to pay fees, and lack of social support to attend appointments. Access was categorized as adequate if the nearest facility was within 5 km and no barriers were reported, moderately accessible if the facility was > 5 km but no other barriers, and limited access if the facility was > 5 km and participants reported ≥ 1 barrier).

To ensure high-quality data collection, all investigators and field workers were thoroughly trained in the study objectives, methodology, and use of the data collection tools. The training also emphasized building rapport, confidentiality, and ethical conduct throughout the data-gathering process. Data were checked regularly in the field for completeness and accuracy. Any unclear or missing responses were verified and corrected in the field itself by revisiting the concerned households.

**Table 1:** Measures used in the study

Variable Type	Variables
Dependent	Multimorbidity (≥ 2 chronic conditions)
Independent	- Health literacy, Short Form Health Literacy Scale (HLS-SF-47)
	- Physical activity, Global Physical Activity Questionnaire (GPAQ)
	- Social support, Multidimensional Scale of Perceived Social Support (MSPSS)
	Quality of life, Short Form-12 (SF-12)
Covariates	
Sociodemographic factors:	-Age, Gender
	- Marital status
	- Education
	- Socioeconomic status (measured by modified BG Prasad scale)
Health behaviors:	Smoking status (categorized as a non-smoker, current smoker, former smoker)
	Alcohol use (drinking frequency and drinks per occasion)
	Dietary patterns (assessed by food frequency questionnaire)
Healthcare access:	• Distance to nearest health facility
	• Barriers to healthcare access (lack of transportation, inability to pay fees, lack of social support to attend appointments)

Table 1 summarizes the key-dependent, independent, and covariate variables that were measured in the study, along with the tools used to assess each variable. The table categorizes the variables into dependent (multimorbidity), independent (health literacy, physical activity, social support, quality of life), and covariates including sociodemographic factors, health behaviors, and healthcare access.

### Data analysis

The collected data were meticulously processed and analyzed using the Statistical Package for Social Sciences (SPSS version 26). Socioeconomic status was measured using the modified BG Prasad classification based on the consumer price index for the study year. This tool classifies SES into upper, upper middle, middle, lower middle, and lower categories based on the monthly per-capita income [22].

The prevalent disease clusters were identified based on the chronic condition combinations reported by the study participants with multimorbidity. The three most frequently occurring combinations were categorized as the common multimorbidity clusters in this population.

The SF-12 scale was analyzed by calculating the physical and mental component summary scores, which range from 0 to 100 with higher scores indicating better health-related quality of life. The physical and mental component scores were computed using standard scoring algorithms and compared between older adults with and without multimorbidity using the independent samples t-test.

Descriptive statistics were used to summarize the prevalence of multimorbidity and the pattern of chronic disease clusters in the study population. Bivariate analyses using t-tests and chi-square tests were conducted to compare health-related quality of life between older adults with and without multimorbidity.”

Multivariable logistic regression analysis was employed to identify factors associated with multimorbidity after adjusting for sociodemographic covariates. The model is represented by:

Log(p/1 − p) = b0 + b1 × 1 + b2 × 2 + …. + bpXp.

Where p is the probability of having multimorbidity, b0 is the constant, b1 to bp are regression coefficients, and X1 to Xp are explanatory variables including health literacy, physical activity, social support, and sociodemographic factors. The significance level was set at *p* < 0.05, ensuring a robust statistical threshold.

### Ethical consideration

This study started after ethical clearance was obtained from the Institutional Ethics Committee. (REF No: 216/03/23). Written Informed consent was obtained first after the purpose of the study was explained, and participants were not obliged to answer any questions they did not like or were free to terminate the interview at any given time. Assurance was given that confidentiality concerning their information would be strictly maintained.

## Results

Table 2 shows the sociodemographic characteristics of the 800 study participants. Frequencies and percentages are presented for the categories of age, sex, religion, marital status, education level, and socioeconomic status (SES).

**Table 2:** Sociodemographic characteristics of the study participants (*n* = 800)

Characteristic	*n*	%
Age (years)		
65–69	420	52.5
70–79	280	35.0
≥ 80	100	12.5
Sex		
Male	400	50.0
Female	400	50.0
Religion		
Hindu	502	62.8
Muslim	198	24.8
Christian	100	12.5
Marital Status		
Married	444	55.5
Widowed	236	29.5
Unmarried	120	15.0
Education		
No formal education	363	45.4
Primary school	277	34.6
Secondary school	100	12.5
Higher secondary/diploma	39	4.9
Graduate and above	21	2.6
SES		
Upper	52	6.5
Upper middle	158	19.8
Middle	260	32.5
Lower middle	180	22.5
Lower	150	18.8
Physical Activity Level		
Active	222	27.8
Moderately active	298	37.3
Inactive	280	35.0
Smoking Status		
Non-smoker	460	57.5
Current smoker	124	15.5
Former smoker	216	27.0
Health care access		
Adequate access	400	50.0
Moderate access	296	37.0
Limited access	104	13.0
Mean Social Support (MSPSS score) (SD)	Mean ± SD: 65.2 ± 12.8	

socioeconomic status (SES)

The prevalence of multimorbidity (defined as ≥ 2 chronic conditions) among the 800 study participants is presented in Table 3. Overall, 500 participants (62.5%) were found to have multimorbidity.

**Table 3:** Prevalence of multimorbidity (≥ 2 chronic conditions) among study participants (*n* = 800)

Multimorbidity	*n* (%)
Absent	300 (37.5%)
Present	500 (62.5%)

Table 4 presents the prevalent disease clusters among older adults with multimorbidity in the urban slum setting. The most common combination was hypertension paired with diabetes, affecting 32% (160 participants) of those with multimorbidity. The second most frequent cluster was hypertension combined with osteoarthritis, observed in 24% (120 participants) of the multimorbid group. Other notable disease combinations included diabetes with heart disease (16%), respiratory disease with heart disease (12%), and depression with osteoarthritis (8%). Less common pairings were diabetes with stroke (4%) and heart disease with cancer (2%). Interestingly, a small proportion (2%) of participants exhibited a triad of conditions: hypertension, diabetes, and osteoarthritis. These findings highlight the complex interplay of chronic conditions in this population, with cardiovascular and metabolic disorders frequently co-occurring. The prevalence of these specific disease clusters underscores the need for integrated care approaches that address multiple chronic conditions simultaneously in older adults residing in urban slums.

**Table 4:** Prevalent disease clusters among older adults with multimorbidity (*n* = 500)

Disease Clusters	*n* (%)
Hypertension + Diabetes	160 (32%)
Hypertension + Osteoarthritis	120 (24%)
Diabetes + Heart disease	80 (16%)
Respiratory disease + Heart disease	60 (12%)
Depression + Osteoarthritis	40 (8%)
Diabetes + Stroke	20 (4%)
Heart disease + Cancer	10 (2%)
Hypertension + Diabetes + Osteoarthritis	10 (2%)

Table 5 presents the comparison of health-related quality of life, as measured by the SF-12 physical and mental component summary scores, and health literacy, as measured by the HLS-SF-47, between older adults with and without multimorbidity. The mean SF-12 physical component score was significantly lower for those with multimorbidity (39.7 ± 6.5) compared to those without multimorbidity (42.5 ± 5.2), with a *p*-value < 0.001. Similarly, the mean SF-12 mental component score was significantly lower for the multimorbidity group (45.3 ± 8.9) versus the non-multimorbidity group (49.2 ± 7.1), with *p* < 0.001. This indicates that the presence of multimorbidity was associated with poorer physical and mental quality of life. For health literacy, the multimorbidity group had a significantly lower mean HLS-SF-47 score (24.7 ± 6.2) than the non-multimorbidity group (32.1 ± 5.8), *p* < 0.001, showing that increased multimorbidity was correlated with lower health literacy. Additionally, the multimorbidity group had significantly lower social support, as measured by lower mean MSPSS scores (60.3 ± 13.5) compared to the non-multimorbidity group (71.5 ± 11.2), *p* < 0.001. Overall, Table 5 highlights the significant negative impact of multimorbidity on health-related quality of life across physical, mental, and social health domains in this older adult urban slum population.

**Table 5:** Comparison of health-related quality of life (SF-12), health literacy (HLS-SF-47), and Social Support (MSPSS scale) between older adults with and without multimorbidity

SF-12 scores	Multimorbidity Absent (*n* = 300)	Multimorbidity Present (*n* = 500)	*P* value
Physical Component Summary	42.5 ± 5.2	39.7 ± 6.5	<0.001 **
Mental Component Summary	49.2 ± 7.1	45.3 ± 8.9	<0.001 **
Mean HLS-SF-47 scale scores	32.1± 5.8	24.7 ± 6.2	< 0.001**
Mean MSPSS score	71.5 ± 11.2	60.3 ± 13.5	< 0.001**

*P* < 0.05; *-significant

Table 6 shows the Pearson correlation between health literacy (HLS-SF-47) and quality-of-life scores on the SF-12 scale.

**Table 6:** Correlation between health-related quality of life and health literacy

	SF-12 Physical Component Score	SF-12 Mental Component Score	HLS-SF-47 Score
SF-12 Physical Component Score	1		
SF-12 Mental Component Score	0.46*	1	
HLS-SF-47 Score	0.52*	0.38*	1

*Correlation is significant at *p* < 0.05

Table 7 shows the Multivariable logistic regression analysis was conducted to identify factors associated with multimorbidity. Older age (per year increase) was associated with greater odds of multimorbidity (AOR 1.05, 95% CI 1.02–1.09). Female gender (AOR 1.86, 95% CI 1.12–3.08), widowed status (AOR 2.05, 95% CI 1.15–3.65), no formal education (AOR 3.12, 95% CI 1.52–6.41), lower socioeconomic status (AOR 2.35, 95% CI 1.22–4.52), being a current smoker (AOR 2.35, 95% CI 1.67–3.46) or former smoker (AOR 2.15, 95% CI 1.59–4.23), physical inactivity (AOR 1.68, 95% CI 1.027–2.77), and lack of social support (AOR 1.57, 95% CI 1.01–2.45) also increased the likelihood of multimorbidity. For every 1 unit increase in the health literacy score, the odds of having multimorbidity decrease by 19% (AOR 0.81, 95% CI 0.78–0.91). Additionally, limited healthcare access was associated with higher odds of multimorbidity (AOR 2.49, 95% CI 1.88–4.27).

**Table 7:** Factors associated with multimorbidity among study participants according to multivariable logistic regression analysis

Variable	Category	Adjusted Odds Ratio (95% CI)	*P* value
Age	Per year increase	1.05 (1.02–1.09)	0.002*
Sex	Male	1 (Reference)	
	Female	1.86 (1.12–3.08)	0.016*
Religion	Hindu	1 (Reference)	
	Muslim	1.23 (0.67–2.77)	0.501
	Christian	1.15(0.59–2.23)	0.683
Marital Status	Married	1 (Reference)	
	Widowed	2.05 (1.15–3.65)	0.015*
Smoking status	Non-smoker	1 (Reference)	
	Current smoker	2.35 (1.67–3.46)	0.04 *
	Former smoker	2.15 (1.59–4.23)	0.03*
Education	Graduate & above	1 (Reference)	
	No formal education	3.12 (1.52–6.41)	0.002*
Socioeconomic Status	Upper	1 (Reference)	
	Lower	2.35 (1.22–4.52)	0.011*
Health Literacy	Per unit increase	0.81(0.78–0.91)	<0.001**
Physical Activity	Active	1 (Reference)	
	Inactive	1.68 (1.02–2.77)	0.04*
Social Support	Adequate	1 (Reference)	
	Lack of Support	1.57 (1.01–2.45)	0.047*
Health care Access	Adequate	1 (Reference)	
	Limited	2.49 (1.88–4.27)	0.015*

*P* < 0.05; *-significant.

In summary, the odds of multimorbidity were positively associated with older age, female sex, being widowed, lower levels of education, smoking, physical inactivity, lack of social support, lack of health literacy, and limited access to healthcare. The analysis highlights the impact of sociodemographic disparities on multimorbidity risk in the study population. Targeted interventions to address modifiable risk factors like health literacy and social support may help reduce the burden of multimorbidity among vulnerable older adults.

## Discussion

In this community-based cross-sectional study, we found a high prevalence of multimorbidity (≥ 2 chronic conditions) affecting more than 60% of older adults residing in urban slum areas. The most prevalent chronic disase were hypertension, diabetes, musculoskeletal disorders, respiratory diseases, and mental health issues. Multimorbidity was significantly associated with lower quality of life, with older adults reporting poorer physical and mental health on the SF-12 scale.

Our findings on the high burden of multimorbidity align with those of previous studies in India, which reported a prevalence ranging from 55 to 65% among community-dwelling older adults in urban slums [23, 24]. The pattern of common chronic conditions observed in this urban slum population also conforms to the epidemiological transition underway in urban regions [25]. With continuing demographic and lifestyle changes, India is facing escalating burdens of noncommunicable diseases manifesting as multimorbidity among its rapidly growing older adult population.

The two most prevalent clusters were hypertension paired with diabetes (in 80 participants, 32%) and hypertension paired with osteoarthritis (in 60 participants, 24%).

These patterns align with multimorbidity data from previous studies in India that also noted hypertension, diabetes, cardiovascular disease, and musculoskeletal disorders as the predominant co-occurring chronic conditions among older adults [26–28].

The high prevalence of certain clusters emphasizes the need to strengthen the integrated screening and management of comorbid conditions such as diabetes and hypertension that tend to coexist and negatively impact outcomes. Tackling common modifiable risk factors and addressing disease combinations through a patient-centered approach can help reduce the burden of multimorbidity as the population ages.

The strong inverse association between multimorbidity and quality of life is consistent with reports across diverse global settings [29, 30]. Managing multiple chronic conditions simultaneously has a detrimental additive effect on physical capacities, psychological well-being, social relationships, and independence in daily living. Multimorbidity also results in complex healthcare needs and polypharmacy, which older adults in resource-constrained slums are ill-equipped to handle. Their poor living conditions, limited access to health services, and lack of social protection exacerbate the challenges of multimorbidity.

A key finding was the high prevalence of inadequate health literacy associated with multimorbidity. After adjusting for sociodemographic variables, the odds ratio of 0.81 indicates that for every 1 unit increase in the health literacy score, the odds of having multimorbidity decrease by 19%. This finding aligns with prior research showing that health literacy is an independent predictor of multimorbidity (31–32). Low health literacy can impede the self-management of chronic diseases, medication adherence, and utilization of preventive services [33]. Enhancing health literacy through community education and capacity building may help reduce the risk and effects of multimorbidity among vulnerable older adult people.

The Multivariable regression analysis showed that inadequate health literacy, lack of physical activity, and lack of social support were significantly associated with a higher likelihood of multimorbidity in this urban slum population. These findings align with prior studies demonstrating the role of health literacy and social determinants in multimorbidity risk. A systematic review found that low health literacy was associated with greater multimorbidity prevalence in several studies [34]. Other research has linked social isolation and poorer social support with an increased number of chronic conditions among older adults [35, 36]. Finally, a cohort study in Brazil concluded that insufficient physical activity was predictive of developing multimorbidity over a 2-year follow-up period [37]. Taken together, these modifiable factors related to health behaviors, capacities, and social environment appear to contribute significantly to the development of multimorbidity, even after accounting for sociodemographic characteristics.

Our study provides representative data on the prevalence of multimorbidity and its impact on quality of life, specifically among older residents of an Indian urban slum - an underserved population often excluded from national health surveys. This highlights the disproportionate multimorbidity burden imposed by socioeconomically marginalized older adult groups dwelling in informal settlements. The much higher prevalence compared to rural counterparts implies urban slum conditions like concentrated poverty, lack of infrastructure, and constrained healthcare access could exacerbate the development of multiple chronic illnesses.

The cumulative out-of-pocket expenditure for healthcare poses catastrophic financial risks to low-income slum households already struggling to meet daily necessities. Older adults with multimorbidity likely face prohibitive barriers in affording treatment and medications over long periods. Many are forced to choose between healthcare costs and other basic needs, which further worsens their disease prognosis and quality of life. Their social vulnerability and lack of financial protection mechanisms make it difficult to effectively manage complex healthcare needs arising from multimorbidity.

Targeted interventions to alleviate the multimorbidity burden among the urban poor should address context-specific social determinants of health-spanning living conditions, access to health services, community awareness, and support systems. Health policies must recognize deprived urban groups and provide tailored financial risk protection alongside prevention and screening initiatives.

Limitations of this study include the cross-sectional design, which restricts causal inference about the association between multimorbidity and quality of life. The reliance on self-reported diagnoses of chronic conditions could result in underreporting. We selected only one slum area, which may limit the generalizability of the findings to other urban slums that differ substantially in their demographic composition and health profiles. Nonetheless, the study provides novel insights into the vulnerability of older slum dwellers to multimorbidity and its adverse effects.

### Recommendations

- Integrated screening and management programs for multimorbidity should be implemented in urban slums targeting older adults.
- Affordable primary care and geriatric services need to be made accessible within slum settings.
- Public health policies and interventions must address social determinants such as education, financial security, and living conditions in slums.
- Family members and caregivers of older adults with multimorbidity require training and support.
- Community awareness of healthy lifestyles, preventive behaviors, and self-care should be created.
- Health literacy of older adults in urban slums should be improved through community education programs.
- Social support systems and financial protection mechanisms are needed for vulnerable older adult groups.

## Conclusion

Multimorbidity among older adults in urban slums requires urgent policy attention and action. A multipronged strategy should focus on both preventive and management aspects, spanning health promotion, community-based screening, affordable primary care, geriatric services, and social assistance. Tackling socioeconomic deprivation alongside lifestyle risks and timely disease management can help reduce the multimorbidity burden and improve the quality of life among marginalized older adult people in urban India. The high prevalence of inadequate health literacy associated with multimorbidity suggests low health awareness and self-care capacities among urban older adult slum dwellers. Targeted interventions to improve health literacy through community outreach, patient education, simplified treatment guidelines, and capacity building of family caregivers are essential.

### Electronic supplementary material

Below is the link to the electronic supplementary material.

Supplementary Material 1

## Data Availability

No datasets were generated or analysed during the current study.

## Abbreviations

HLS-SF-47: Short Form Health Literacy Scale
SF-12: Short Form-12
