# Supplementary material for: Multimorbidity, health Literacy, and quality of life among older adults in an urban slum in India: a community-based cross-sectional study
Source: BMC Public Health. 2024 Jul 9;24:1833. doi: 10.1186/s12889-024-19343-7 (PMC11234527; doi:10.1186/s12889-024-19343-7)
Supplement: Supplementary file 1 — Supplementary Material 1 [file 12889_2024_19343_MOESM1_ESM.docx]

Supplementary File 1: Questionnaire

Section 1: Sociodemographic Information

1. Age (in completed years):
2. Gender: a) Male b) Female
3. Religion: a) Hindu
   b) Muslim c) Christian d) Other
4. Marital status: a) Married b) Widowed c) Separated/Divorced d) Never married
5. Education level: a) No formal education b) Primary school c) Middle school d) High school e) Intermediate/Diploma f) Graduate and above
6. Occupation: a) Unemployed b) Self-employed c) Salaried employment d) Daily wage labor e) Retired
7. Monthly household income: a) Less than Rs. 5000 b) Rs. 5001 - Rs. 10,000 c) Rs. 10,001 - Rs. 20,000 d) More than Rs. 20,000
8. Living arrangements: a) Living alone b) Living with spouse c) Living with children d) Living with other relatives

Section 2: Health Status and Behaviors

1. Do you currently have any of the following chronic health conditions?

a) Hypertension b) Diabetes c) Heart disease d) Stroke e) Chronic respiratory disease f) Arthritis/joint pain g) Cancer h) Mental health condition i) None

1. How many prescription medications do you take daily?
2. How would you rate your current health status?

a) Excellent b) Very good c) Good d) Fair e) Poor

1. Do you currently smoke tobacco?

a) Yes b) No c) Former smoker

1. Do you currently drink alcohol?
2. Yes b) No c) Former drinker
3. What's the distance to the nearest health facility?

Section 3: Health Literacy (HLS-SF-47 scale)

Here are sample sections with the HLS-SF-47 and SF-12 scale questions:

Section 3: Health Literacy (HLS-SF-47 scale)

Response options:

1 - Never

2 - Rarely

3 - Sometimes

4 - Often

5 - Always

1. I feel confident using information from health resources to make health decisions.

2. I ask healthcare providers about health resources that are available to me.

3. I make health decisions based on information I find in health resources.

4. I have someone available to help me read health information if I need assistance.

5. I have access to reliable transportation to health services if needed.

6. I have access to quality health care services if I need them.

7. I feel I have good health insurance coverage that meets my needs.

8. I am able to pay for health care services that are not covered by my health insurance.

9. I am able to speak and listen effectively during medical appointments.

10. I am able to ask my healthcare providers questions to get the health information I need.

11. I collect health information from many different sources.

12. I compare health information from different sources.

13. I critically analyze the health information I find.

14. I feel confident determining the quality of health information.

15. I ask healthcare providers about the quality of health information I find.

16. I have the skills I need to evaluate the reliability of health resources.

17. I can tell high quality from low quality health resources.

18. I feel confident I can tell if health information is right or wrong.

19. I have someone available who can help me understand health information if I need it.

20. I have the knowledge I need to manage complex health needs.

21. I know what questions to ask healthcare providers to get the information I need.

22. I have access to credible health services and providers when needed.

23. I have access to health care providers who communicate clearly with me.

24. The health care providers I see listen carefully to my concerns.

25. I feel comfortable telling a healthcare provider when I disagree with them.

26. I make sure I fully understand my healthcare providers' instructions.

27. I am able to have open discussions about my health with providers.

28. I discuss personal or family problems that may affect my health with providers.

29. I make sure healthcare providers understand my situation before making recommendations.

30. I bring a list of questions when meeting with healthcare providers.

31. I bring someone who can help me communicate with providers if needed.

32. I prepare questions before a medical appointment.

33. I have ready access to credible health information resources.

34. I have access to culturally and linguistically appropriate health services.

35. I have access to health care providers who understand my culture and values.

36. I have access to health services without facing discrimination.

37. I accurately follow the health providers' instructions.

38. I read and understand all health-related information that healthcare providers give me.

39. I understand the risks and benefits of treatment options available to me.

40. I make sure that healthcare providers understand my concerns.

41. I feel I can manage my health even when stressed.

42. I avoid health behaviors that negatively affect my health.

43. I do things that positively affect my health.

44. I pay attention to my health and make changes when necessary.

45. I set goals for my health and fitness.

46. I look for resources and support to improve my health.

47. I make time to be physically active.

Section 4: Quality of Life (SF-12 scale)

Response options:

1 - Excellent

2 - Very good

3 - Good

4 - Fair

5 - Poor

1. In general, would you say your health is:

2. During the past 4 weeks, how much did physical health problems limit your physical activities (such as walking or climbing stairs)?

3. During the past 4 weeks, how much difficulty did you have doing your daily work, both at home and away from home, because of your physical health?

4. How much bodily pain have you had during the past 4 weeks?

5. During the past 4 weeks, how much energy did you have?

6. During the past 4 weeks, how much did your physical health or emotional problems limit your usual social activities with family or friends?

7. During the past 4 weeks, how much have you been bothered by emotional problems (such as feeling anxious, depressed or irritable)?

8. During the past 4 weeks, how much did personal or emotional problems keep you from doing your usual work, school or other daily activities?

9. During the past 4 weeks, how would you rate your mental health in general?

10. During the past 4 weeks, how satisfied have you been with your social activities and relationships?

11. During the past 4 weeks, how would you rate your satisfaction with your ability to perform regular daily activities?

1. During the past 4 weeks, how would you rate your satisfaction with your ability to work (include work at home)?

Section 5: Social Support (MSPSS Scale)

Instructions: We are interested in how you feel about the following statements. Read each statement carefully. Indicate how you feel about each statement.

Response options:

1 - Very strongly disagree

2 - Strongly disagree

3 - Mildly disagree

4 - Neutral

5 - Mildly agree

6 - Strongly agree

7 - Very strongly agree

There is a special person who is around when I am in need.

There is a special person with whom I can share my joys and sorrows.

My family really tries to help me.

I get the emotional help and support I need from my family.

I have a special person who is a real source of comfort to me.

My friends really try to help me.

I can count on my friends when things go wrong.

I can talk about my problems with my family.

I have friends with whom I can share my joys and sorrows.

There is a special person in my life who cares about my feelings.

My family is willing to help me make decisions.

I can talk about my problems with my friends.

Section 6: Physical Activity (GPAQ Scale)

Instructions: Now I am going to ask you about the time you spend doing different types of physical activity in a typical week. Please answer these questions even if you do not consider yourself to be a physically active person.

Think first about the time you spend doing work. Think of work as the things that you have to do such as paid or unpaid work, studying/training, household chores, harvesting food/crops, fishing or hunting for food, seeking employment.

In the last 7 days, on how many days did you do vigorous work activities like heavy lifting, digging, construction work, or climbing?

How much time did you usually spend on one of those days doing vigorous work activities?

_____ hours ____ minutes

In the last 7 days, on how many days did you do moderate work activities like carrying light loads or sweeping?

How much time did you usually spend on one of those days doing moderate work activities?

____ hours ____ minutes

In the last 7 days, on how many days did you walk or bicycle for at least 10 minutes continuously to get to and from places?

How much time did you usually spend on one of those days to walk or bicycle to get to and from places?

____ hours ____ minutes

In the last 7 days, on how many days did you do moderate recreational activities like bicycling at a regular pace, swimming at a regular pace, and doubles tennis?

How much time did you usually spend on one of those days doing moderate recreational activities?

___ hours ___minutes

In the last 7 days, on how many days did you do vigorous recreational activities like aerobics, running, fast bicycling, or fast swimming?

How much time did you usually spend on one of those days doing vigorous recreational activities?

____ hours ___ minutes

This covers key components of the GPAQ to assess physical activity at work, for transport, and recreation.
